# Exploring the Impact of health-care cost sharing mechanisms in health service users for all the 27 member countries of the EU

**DOI:** 10.1186/2052-3211-8-S1-P29

**Published:** 2015-10-05

**Authors:** Andreas Siakou, Costas Athanasakis

**Affiliations:** 1 Faculty of Economics and Management, Health Policy and Planning, Open University of Cyprus, Nicosia, 2252, Cyprus

## Objectives

The main objective of this study was to investigate the cost-sharing mechanisms in pharmaceutical expenditure in EU-27 and to further explore the impact of policies on the broader NHS. The study was based on data from February to March 2015.

## Methods

The following were the main research questions addressed: What are the main differences among the individual case studies, which were implemented in the countries concerned? Are the results included in this study expected to have a positive or negative impact on health care users? Are these results expected to ensure equal proportional allocation of the cost-sharing mechanisms among health care users?

Regarding the methodology of the study, an extensive analysis of the targeted policies and practices was carried out based on the data obtained from the databases of the OECD, PubMed and Google Scholar.

## Results

The results showed that, in the European South cost-sharing was introduced and implemented as an austerity measure in the context of an imposed fiscal discipline, with Germany being the auditor of the European Central Bank. On the other hand, in the European North cost-sharing has traditionally been an integral part of their NHS. This cost-sharing policy has lead to significant impacts on health care systems, such as the control of excess demand and health services as well as the control of moral hazard. Cost-sharing policy has affected users as well. For instance, compensation issues in the case of biosimilars have not yet been resolved and the rate of visits in non-governmental organizations and other social clinics has increased tremendously (up to 23 %).

## Conclusions

Overall, the implementation of a strong cost-sharing mechanism can facilitate a sustainable and efficient NHS system based on the principle of equal rights. So far, all changes within NHS systems were proved necessary for ensuring viability and sustainable development. This policy appears to be a step in the right direction for improving the scope and the quality of the services offered by the NHS.

